# On the need for integrating cancer into the One Health perspective

**DOI:** 10.1111/eva.13303

**Published:** 2021-10-08

**Authors:** Antoine M. Dujon, Joel S. Brown, Delphine Destoumieux‐Garzón, Marion Vittecoq, Rodrigo Hamede, Aurélie Tasiemski, Justine Boutry, Sophie Tissot, Catherine Alix‐Panabieres, Pascal Pujol, François Renaud, Frédéric Simard, Benjamin Roche, Beata Ujvari, Frédéric Thomas

**Affiliations:** ^1^ CREEC/CANECEV (CREES) Montpellier France; ^2^ MIVEGEC Université de Montpellier, CNRS, IRD Montpellier France; ^3^ School of Life and Environmental Sciences Centre for Integrative Ecology Deakin University Waurn Ponds Vic. Australia; ^4^ Department of Integrated Mathematical Oncology Moffitt Cancer Center Tampa Florida USA; ^5^ IHPE Université de Montpellier CNRS Ifremer Université de Perpignan Via Domitia Montpellier France; ^6^ Tour du Valat Research Institute for the Conservation of Mediterranean Wetlands Arles France; ^7^ School of Natural Sciences University of Tasmania Hobart Tas. Australia; ^8^ Univ. Lille CNRS Inserm CHU Lille Institut Pasteur de Lille U1019‐UMR9017‐CIIL‐Centre d'Infection et d'Immunité de Lille Lille France; ^9^ Laboratory of Rare Human Circulating Cells (LCCRH) University Medical Centre of Montpellier Montpellier France; ^10^ Oncogenetic Department University Medical Centre of Montpellier Montpellier France

**Keywords:** cancer, comparative oncology, infectious diseases, One Health, Peto’s paradox

## Abstract

Recent pandemics have highlighted the urgency to connect disciplines studying animal, human, and environment health, that is, the “One Health” concept. The One Health approach takes a holistic view of health, but it has largely focused on zoonotic diseases while not addressing oncogenic processes. We argue that cancers should be an additional key focus in the One Health approach based on three factors that add to the well‐documented impact of humans on the natural environment and its implications on cancer emergence. First, human activities are oncogenic to other animals, exacerbating the dynamics of oncogenesis, causing immunosuppressive disorders in wildlife with effects on host–pathogen interactions, and eventually facilitating pathogen spillovers. Second, the emergence of transmissible cancers in animal species (including humans) has the potential to accelerate biodiversity loss across ecosystems and to become pandemic. It is crucial to understand why, how, and when transmissible cancers emerge and spread. Third, translating knowledge of tumor suppressor mechanisms found across the Animal Kingdom to human health offers novel insights into cancer prevention and treatment strategies.

## 1 INTRODUCTION

The “One World–One Health” concept, initiated in 2004, recognizes the close connections between health of humans and that of other animals and our shared environment. The importance of this concept is evidenced by the accelerated emergence or re‐emergence of zoonoses (e.g., Ebola, current COVID‐19 pandemic) over the last decades (Peyre et al., 2021). Although the One Health approach proposes a holistic view of health, it has, and still remains, largely focused on zoonotic and vector‐borne diseases. Reasons behind this bias are multiple, ranging from the fact that historically it has originated from a collaboration between human and veterinary medicines, and also because zoonotic diseases undoubtedly represent major health and economic threats to human societies. The scope has broadened with the inclusion of other fields, such as antimicrobial resistance (WHO, 2015), (eco)toxicology, and health in urban environments (Destoumieux‐Garzón et al., 2018), but not cancer.

The impact of human activities on the natural environment and their implications on cancer emergence in humans have been largely documented (e.g., anthropogenic pollution, occupation, food, and lifestyle; Aktipis & Nesse, 2013) (Briffa et al., 2020; Erren et al., 2015; Turner et al., 2020). These effects and their consequences could increase in the context of climate change (Hiatt & Beyeler, 2020). Beyond this acknowledged link between the environment and cancer, we discuss here three reasons for a broader inclusion of cancer into the One Health concept.

First, the interplay/interactions between cancer and infectious diseases make arduous to separate these communicable and non‐communicable diseases, and their combination could pose higher risk for human and animal health. For instance, cancers can cause immunosuppression 2020); cancers can also be induced by pathogens (Ewald & Swain Ewald, 2015; Jacqueline et al., 2017). However, little attention has been paid to the role of human‐induced cancers in infectious diseases’ emergence and spread. Human activities elevate cancer incidences for wildlife species (via chemical pollution, changes in diet, and reductions in genetic diversity; Baines et al., 2021; Giraudeau et al., 2018; Sepp et al., 2019; Ujvari et al., 2018). As in humans, cancer burdens generate a range of immunosuppressive disorders in livestock, and likely in wildlife (Pollock & Roth, 1989; Vittecoq et al., 2013). Animals with cancer, human or otherwise, may be more susceptible, contagious, and debilitated by infectious diseases. Therefore, the evolutionary mismatch between enhanced human‐induced cancer risks and maladapted cancer defense levels in animals will indirectly amplify pathogen dynamics in wildlife, in both natural and human‐dominated ecosystems. In undisturbed natural environments, a fraction of pathogens is generally maintained by the presence of immunocompromised individuals, a stable equilibrium, that has been reached over evolutionary time (Hassell et al., 2021). By accelerating cancer incidence and the dynamics of oncogenesis, anthropogenic activities can disrupt these equilibria. These altered dynamics are complex and not merely correlated with pollution levels, but also driven by, for example, the different susceptibilities of exposed species to carcinogenesis. For instance, a benthic lifestyle within fish communities contributes to elevated cancer incidence through chronic exposure to contaminated sediment and/or consumption of contaminated invertebrates (Black & Baumann, 1991; Martineau et al., 2002). The consequences of these disturbances are difficult to predict given the cascade of indirect effects that may subsequently occur. For example, the impact of higher cancer incidences and carcinogens will depend on the species most affected by cancer (e.g., predators or prey species) and the resulting immunosuppressive disorders (see, for instance, Perret et al., 2020). The relationship between an animal's immunosuppression and cancer burden (e.g., linear, exponential, negative binomial, and with or without critical thresholds) may also vary with species and/or with the organ in which malignancies develop. Clearly, the roles of pollution‐induced oncogenesis and immunosuppression in boosting pathogen communities in wildlife and their spread to humans are poorly understood at the moment. Given that parasitism and disease spread may also be limited in ecological communities via mechanisms such as the dilution effect (Civitello et al., 2015), pollution‐induced oncogenesis could amplify these transfers if it concomitantly promotes a decline in biodiversity.

A second reason for elevating the importance of cancers in the One Health approach concerns cancers that can themselves become transmissible. There are currently nine known transmissible cancer lineages (one in dogs, two in Tasmanian devil populations, and six in marine bivalves; Dujon, Bramwell, et al., 2020; Dujon, Gatenby, et al., 2020), some of which threaten the survival of animal populations (and species) and are detrimental to ecosystem stability (see, for instance, Hamede et al., 2020). Indubitably, this number is a gross underestimate due to poor monitoring of cancer in wildlife (Dujon, Bramwell, et al., 2020; Dujon, Gatenby, et al., 2020; Dujon, Ujvari, et al., 2020; Ujvari et al., 2016). The conditions for the emergence of transmissible cancers, while poorly understood, require a perfect storm of sequential steps that is reminiscent of a parasitic lifestyle. First, the tumor must shed a large number of cells that are able to survive outside of or on the surface of the original host. Those cells, when in contact with a new host, must then evade immune recognition (particularly challenging in vertebrates) and successfully proliferate in the correct tissue of the new host (Ujvari et al., 2017). Environmental stress, lack of genetic diversity within the host population, and other infectious diseases may weaken the host's immune system enabling transmission and establishment of cancer cells from one individual to another (Dujon, Bramwell, et al., 2020; Dujon, Gatenby, et al., 2020; Dujon, Ujvari, et al., 2020). Human activities may also disperse certain transmissible cancers across the globe, giving previously isolated outbreaks pandemic potentials. Already, a single transmissible cancer lineage has been observed in multiple marine mussel species across multiple oceans, suggesting dispersal by aquaculture and/or shipping (Bramwell et al., 2021; Skazina et al., 2021; Yonemitsu et al., 2019). A full understanding of the ecological impacts of transmissible cancers on ecosystems and biodiversity will require integrating tools from landscape ecology, conservation biology, and epidemiology to identify wildlife species at risk of contagious malignant cell lines, detect their emergence, anticipate their spread, and evaluate consequences to natural and human‐dominated ecosystems (Bramwell et al., 2021; Dujon et al., 2020; Dujon, Gatenby, et al., 2020; Dujon, Ujvari, et al., 2020). A worst‐case scenario might involve the emergence of a communicable cancer in humans or in livestock, yielding environmental and human/animal health crises similar to classical epidemics and epizootics.

Finally, a third reason to consider cancer in the One Health approach lies in the scientific insights provided by comparative oncology focusing on the diverse tumor suppression mechanisms that have evolved across the animal kingdom (Nunney et al., 2015; Schiffman & Breen, 2015). Cancers appeared during the transition from unicellular to metazoan life, approximately one billion years ago (Domazet‐Lošo & Tautz, 2010). Cancers occur in all branches of multicellular life, and they have evolved diverse adaptations to prevent cancer initiation, suppress malignant progression, and alleviate negative fitness consequences of tumor burdens (Aktipis et al., 2015). For instance, one might expect large/long‐lived animals to have more cancers than smaller/shorter‐lived ones, given that the former have many more cell divisions that potential give rise to cancer‐initiating mutations, but such a relationship across species is not observed, due to the various mechanisms of cancer resistance that have evolved in large and long‐lived species (i.e., Peto's Paradox, Tollis et al., 2017). Even in smaller species, under particular ecological conditions, selection can favor high levels of cancer resistance, as observed in naked‐mole rats and bats (Lambert & Portfors, 2017; Seluanov et al., 2018; Tian et al., 2013). Scientists are increasingly focusing on the ways natural selection has protected animal species from cancer (Boutry et al., 2020). This work is deciphering the underlying tissue, cellular, and genomic mechanisms in cancer‐resistant/tolerant species (Abegglen et al., 2015; Keane et al., 2015; Sulak et al., 2016). Another particularly interesting direction of comparative oncology investigates the local evolution of adaptations in species or populations that have been living in environments with excesses of naturally occurring carcinogens (Hourdez et al., 2021; Vittecoq et al., 2018). To what extent do these populations show adaptations to prevent the emergence of cancer versus adaptations to mitigate the negative effects of cancer burdens? To what extent do these populations’ anti‐cancer adaptations increase or decrease susceptibility to infectious diseases? Of particular interest would be adaptations that simultaneously reduce cancer and infections. Studying species and ecological communities chronically exposed to carcinogens, with a particular focus on host–pathogen dynamics, could shed light on the possible impacts and repercussions of human‐induced cancers within wildlife species (Vittecoq et al., 2018). From a medical perspective, the adaptations of long‐lived mammals and other species for preventing and suppressing cancer should inspire nature‐based solutions to cancer, such as novel cancer treatment strategies, by mimicking the processes allowing these organisms to prevent or limit malignant progression despite high levels of mutagenic substances. Thus, comparative oncology offers key insights into cancer epidemiology, prevention, and novel therapies for humans.

**FIGURE 1:**
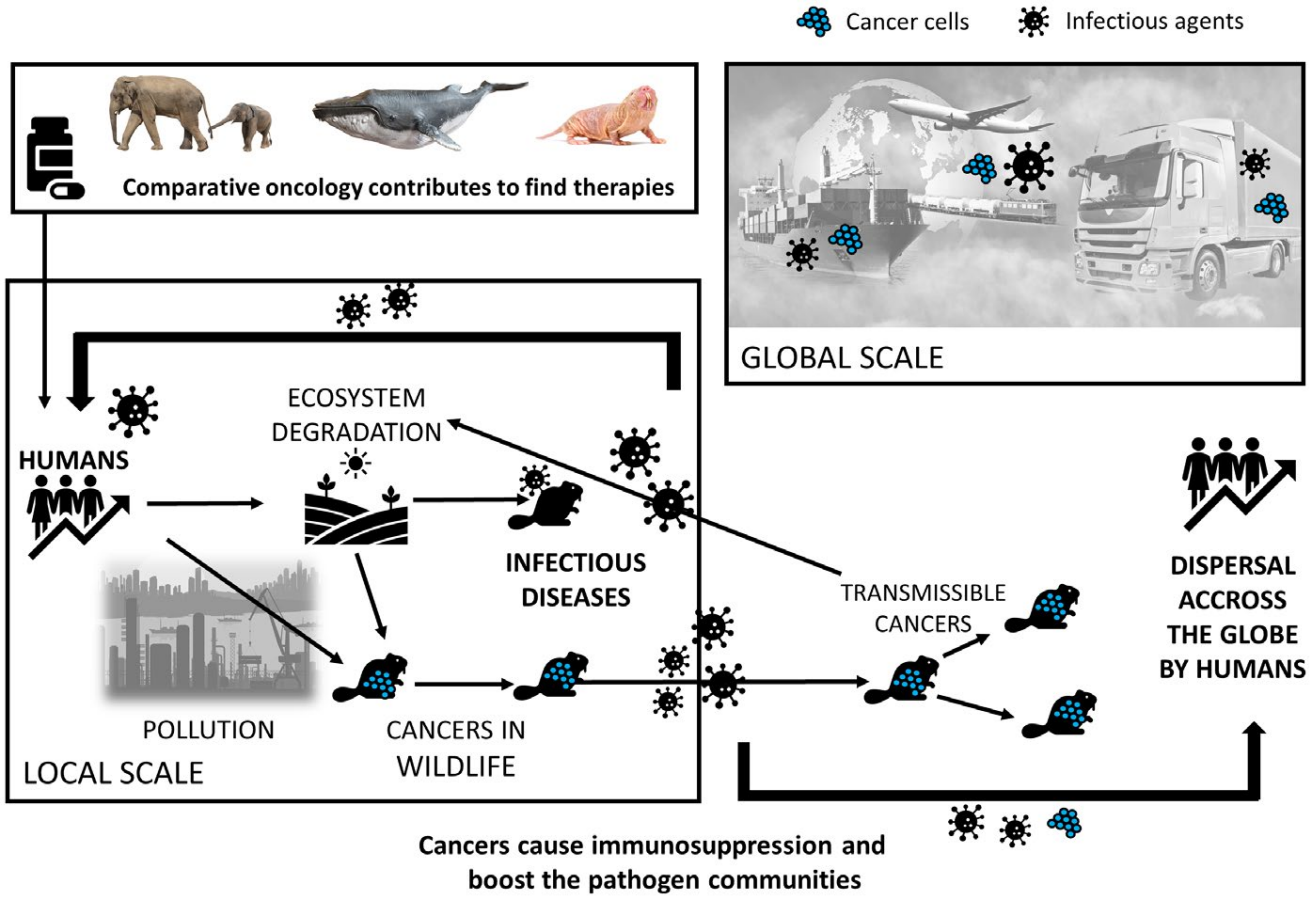
Cancer in the One Health perspective: comparative oncology, interplay between human activities, novel cancer risks experienced by wildlife species, and infectious disease dynamics

In conclusion, humans, domestic animals, and wildlife species are on the front lines of environmental changes and exposure to toxic hazards from anthropogenic activities. While it is well recognized that these processes directly affect immunity and hence pathogen dynamics (e.g., Dittmar et al., 2014; Tracy et al., 2020), much less is known concerning the indirect links resulting from interactions involving non‐communicable diseases, such as cancer. The direct and indirect effects do not necessarily yield the same outcomes. Cancer‐causing or cancer‐associated processes are undoubtedly an underappreciated health consequence of modern anthropogenic changes, and they cannot be considered in isolation from other biological players in ecosystems, especially pathogens. Ecological impacts, sooner or later, are usually followed by evolutionary responses, leading to novel equilibria in ecosystems. However, we currently ignore how the novel equilibria will be achieved and shaped in an increasingly stressful world. A particular question remaining to be answered is how pollution and consequent oncogenic processes will impact infectious diseases and thus human health particularly before reaching a novel equilibrium. Exploring the interplay between human activities, novel cancer risks experienced by wildlife species, cancer defense evolution, biotic interactions, and infectious disease dynamics, notably those transmissible to humans, should become a stronger topic within the One Health perspective (Figure 1). Because one single method or model cannot thoroughly describe these complex dynamics, more than ever researchers must engage in greater exchanges and collaborations involving different disciplines, such as field and experimental ecology, epidemiology, eco‐toxicology, immunology, evolutionary biology, oncology, and pharmacy.

## Data Availability

Data sharing is not applicable to this article as no new data were created or analyzed in this study.
